# Attitude of Thai Dental Practitioners towards the Use of Botulinum Toxin in Dentistry

**DOI:** 10.3390/ijerph19031878

**Published:** 2022-02-08

**Authors:** Sasi Laorpipat, Pornpoj Fuangtharnthip, Suraphong Yuma, Chanita Tantipoj

**Affiliations:** 1 Department of Advanced General Dentistry, Faculty of Dentistry, Mahidol University, Ratchathewi, Bangkok 10400, Thailand; sasi.lao@mahidol.ac.th (S.L.); pornpoj.fun@mahidol.ac.th (P.F.); 2 Department of Physics, Faculty of Science, Mahidol University, Ratchathewi, Bangkok 10400, Thailand; suraphong.yum@mahidol.edu

**Keywords:** botulinum toxin, knowledge, attitude, dentistry

## Abstract

This study aims to investigate the attitude of Thai dentists towards the use of botulinum toxin (BTX) in dentistry and the associated factors. An online survey was conducted using a semi-structured questionnaire consisting of four parts: demographic data, background knowledge, attitude, and an open-ended question for further suggestions related to BTX usage in dental patients. Multivariate logistic regression was used to analyze factors that affect the decision to use BTX in dentistry, and a content analysis approach was used to describe open-ended suggestion data. We received 444 responses from currently practicing dentists throughout Thailand. Roughly 80% of the participants agreed to the use of BTX in their patients. Most participants were aware that BTX could be used for facial esthetic repairs and bruxism treatment but unaware of other therapeutic benefits. Despite impressively positive attitudes towards BTX use, only 5.9% of the participants had the experience of using BTX in their dental patients. The limit on BTX use is mainly due to the lack of knowledge of related laws and educational resources. In summary, official training courses should be established to promote the safe and legal use of BTX in dentistry in Thailand.

## 1. Introduction

Botulinum toxin, abbreviated to BTX, extracted from *Clostridium botulinum* bacteria, has a paralytic effect that inhibits the release of acetylcholine at the neuromuscular junction [1]. BTX has been used medicinally to treat various ailments, including inappropriate muscle contraction and autonomic disorders, such as hyperhidrosis, sialorrhea, and hypersalivation, and for cosmetic applications, such as treating glabellar lines, crow’s feet, and forehead lines. Recently, it has been used to treat myofascial pain syndrome, tension headaches, and migraines as well [2].

Due to the medical benefits, BTX can be applied in dentistry. It can be used to treat or alleviate the symptoms of dental-related diseases in various ways, such as treating diseases of the jaw muscles and facial pain, helping differential diagnosis, and promoting other dental treatments. In addition, it can also be used to adjust soft tissue to improve esthetics [3]. BTX was first used to treat temporomandibular disorder (TMD) in 1999. BTX is also recommended as an alternative treatment for TMD associated with muscle hyperactivity [4]. BTX has various advantages in treating bruxism, such as reducing the frequency of bruxism and the pain levels and grinding force caused by bruxism, and improving patients’ quality of life [5]. BTX can also be used to correct gummy smile caused by hyperfunction of the upper lip-lifting muscle groups levator labii superioris, levator labii superioris alaeque nasii, zygomaticus major, zygomaticus minor, and depressor septii [6]. The BTX injection depends on the individual’s symptoms and their severity. It was found that BTX injections were effective for creating esthetic satisfaction and patients wanted to repeat this therapy [7]. The BTX’s ability to reduce masticatory muscle activity can help promote other dental treatments, such as helping oral muscles adjust to dentures more easily, reducing relapse caused by spasm of the mentalis muscle after orthodontic treatment, promoting osseointegration of the implant [8], etc. Clearly, BTX has many other benefits aside from just the esthetic aspects.

Currently, the number of dentists who use BTX to treat patients is increasing, and it is widely accepted in many countries around the world. For example, the states of Massachusetts and New Jersey, USA, stipulate that dentists must undergo accredited training courses in order to treat patients with BTX [9]. The British Association of Cosmetic Dental Professionals (BACDP) ruled that dentists can prescribe and inject BTX to treat diseases caused by abnormal muscle function or promote other dental-related therapies, such as osseointegration of implants, while prohibiting misleading advertisement [10]. The Dental Council of Ireland allows dentists to use BTX when treating oral conditions such as deformity of the jaw joint or chronic pain [11]. Similar permission for BTX use has been announced by the Australian Dental Council [12]. In Asia, the Dental Council of Singapore has authorized trained and certified dentists to inject BTX for cosmetic purposes and expanded the practice range of maxillofacial surgeons to be able to treat the head and neck areas [13].

The use of BTX is an optional treatment in combination with conventional dental therapy and is likely to increase in the future. However, the current use of BTX by Thai dentists is still very limited. In this study, we investigated the awareness and attitude of dentists currently practicing in Thailand towards the use of BTX in dentistry to identify the problems or obstacles that prevent Thai dentists from using BTX in dental treatment.

## 2. Materials and Methods

This study is a cross-sectional study approved by the Ethics Committee of Human Research, Faculty of Dentistry and Faculty of Pharmacy, Mahidol University, Bangkok, Thailand (no. MU-DT/PY-IRB 2020/038.2608). Data collection was carried out from December 2020 to April 2021. The participants are dentists who are currently practicing in Thailand with Thai dental licenses. The sample size of at least 384 participants was estimated to represent all 8043 currently practicing dentists in Thailand [14] by adopting the formula for the cross-sectional descriptive study at a 95% confidence interval [15].

The survey was taken on a voluntary basis and conducted by using Google Forms, an online survey platform operated by Google LLC. The questionnaire was developed to reconcile with the dental situation in Thailand. The questionnaire was first evaluated by three dentists who are experts in different fields: oral and maxillofacial surgeons, orofacial pain, and dental education. The questionnaire-evaluating processes were not limited only to examining the importance and usefulness of questions for the study goal, but the accuracy and clarity of the questionnaire were also investigated. We only used the questions with an index of item objective congruence (IOC) larger than 0.5 [16]. The pilot survey was conducted on 12 dental practitioners with academic and dental backgrounds similar to the targeted participants. The internal and external consistency of the questionnaire was evaluated with Cronbach’s alpha coefficients of 0.72 [17] and correlation coefficient of 0.60 [18], respectively. We further improved the clarity of the questions by interviewing these dentists about their understanding of each individual question. The completed questionnaire was later sent to dentists in various hospitals throughout the country via electronic discussion groups (LINE messaging application operated by Line Corporation). The survey data were only obtained directly from those who were willing to answer.

The final version of the questionnaire was divided into 4 parts. The first part was about demography, job-related information, and the BTX-using experience of the participants. The second part assessed background knowledge about the use of BTX in dentistry. The results from both parts were used to determine the potential association of each variable with the attitude of the respondents towards the use of BTX in dentistry, which was evaluated in the third part. The attitude was assessed by using a 5-point Likert scale ranging from 1 for the most negative (i.e., very unimportant, absolutely useless, or strongly disagree) to 5 for the most positive answers (i.e., very important, very useful, or strongly agree). The last part was an open-ended question asking for further suggestions about BTX use in a Thai dental setting.

Statistical analysis was conducted separately for each part according to the questionnaire. To obtain an overview of the survey data, descriptive statistics were used to analyze parts 1–3 based on the frequencies and percentages of each answer. The Pearson’s chi-square or Fisher’s exact test was used to evaluate the correlations among demographic data, knowledge, and the agreement of BTX use in dental patients at a significant level of 0.05. In the second part, we used a dichotomous scale (correct/incorrect) to examine the background knowledge and classified the participants into 2 groups according to the number of correct answers. Out of ten questions, the high-level group was for the participants who got equal to or more than 6 correct answers, whereas the low-level group was for those who were mostly wrong [19]. In part 4 of the questionnaire, the qualitative content analysis approach was used to quantify and describe themes presented in the open-ended suggestion data.

In addition to the descriptive statistics, we assessed the attitude of the participants by calculating the mean score from the 1–5 response scale and defining the favorable outcome as a response scale of 4 and 5. Multiple logistic regression analysis was performed to identify the variables associated with agreeing to the use of BTX in dental patients. Factors were selected with a backward stepwise method. Adjusted odds ratios (AOR) and their 95% confidence intervals (CIs) were used to quantify the associations between variables. All analyses were computed by using PASW Statistics for Windows version 18.0 (SPSS Inc., Chicago, IL, USA).

## 3. Results

The final number of participants was 444 dentists after we excluded 18 participants who were no longer practicing dentistry in Thailand and practiced for less than one year. Table 1 depicts the demographic data of all the respondents, consisting of 121 males (27.25%) and 323 females (72.75%). Seventy-seven percent of all respondents were younger than 35 years old, with an average age of 32.7 years old. A majority of participants (284), comprising 64%, were specialists, while the rest were general practitioners. We further categorized the specialty fields of those 284 specialists into restorative and esthetic dentistry and others. We used italic letters for the specialty in Table 1 as it does not represent all participants of this study. The 169 (59.5%) specialists in restorative and esthetic dentistry include operative specialists, prosthodontists, advanced general dentistry specialists, esthetic dentistry specialists, and dental implantologists. The remaining 115 (40.5%) were other specialists, such as endodontists, oral medicine specialists, periodontists, occlusion and orofacial pain specialists, and oral and maxillofacial surgery specialists. We investigated whether the participants were interested in the field of restorative and esthetic dentistry and found almost half of the participants (49.1%) were interested in this field.

According to Table 1, most of the respondents (59.9%) were working in rural areas, whereas the remaining 40.1% were in Bangkok and its perimeter. A majority of the participants (71.8%) was working at public hospitals. The working experience was consistent with the average age in that most of the participants (90.5%) had an experience of fewer than 20 years. In addition, only 163 participants (36.7%) had experience with BTX. Among them, only 26 participants (16%) had experience treating their patients with BTX. Table 1 also shows the opinion on whether or not the participants agree with using BTX in dental treatment. By using the Pearson’s chi-square and Fisher exact test, many characteristics were found to be statistically associated with agreeing with the use of BTX in dentistry at a *p*-value of less than 0.05 (asterisks in Table 1). Association between the demographics of participants and their opinion on the use of BTX is further discussed in the next section.

Table 2 presents the background knowledge of the participants related to BTX application in dentistry. All ‘yes’ answers in Table 2 correspond to the correct knowledge. The majority of the dentists knew that BTX could be used to help repair facial esthetics, i.e., gummy smile at 70%, masseteric muscle hypertrophy at 66.9%, and bruxism at 75.2%. However, only half of the participants were aware that BTX has other therapeutic benefits such as the treatment of mandibular spasm (52.9%) and myofascial pain (49.8%). Moreover, most of the participants were not aware of the benefits of using BTX to treat trigeminal neuralgia (23.6%) and sialorrhea (15.1%).

We classified the knowledge level of the participants into two levels based on the number of correct answers: high level with 6–10 correct answers and low level with 1–5 correct answers. Only 27.5% of the participants had high-level knowledge, which was associated with an opinion agreeing to the use of BTX in dentistry, also shown in Table 2 at *p* < 0.001 significant level. We found that agreeing to the use of BTX in dental treatment was statistically associated with the BTX knowledge related to repair facial esthetics (gummy smile and masseteric hypertrophy), bruxism, and promoting prosthodontic and orthodontic treatment.

Table 3 lists the questions intended to explore the attitude of the dentists towards the use of BTX in dentistry. In this section, we focus on the percentage of the favorable score (levels 4 and 5). Most of the participants (89.6%) agreed that the BTX could be used as a combination treatment for patients who receive dental services, with an average score of 4.24 (A1 in Table 3). The participants thought that the use of BTX would be beneficial to the dental patients at roughly the same percentage (87.8% in A2). We also asked the respondents about factors that may obstruct BTX use in dental patients. The most concerning point was the lack of knowledge and experience in using BTX (81.9% in A3.2). The second largest obstacle, in the participants’ opinions, was the ambiguity of laws related to the working scope of dentists and the lack of additional educational resources for dentists, sharing approximately the same percentages at 79.7% and 79.3%, respectively. In contrast, increasing costs and inappropriate treatment sites were less concerning, at 57.7% and 55.2%, respectively. From question A4 in Table 3, almost all dentists considered that the use of BTX in dental patients could update their knowledge (93.7%) and improve the treatment efficiency (94.9%). Impressively, 79.3% of the participating dentists were ready to use BTX in dental patients if they had no obstacles (A5).

In the last part of the questionnaire, 167 participants kindly provided a comment or recommendation for the use of BTX in dentistry. By using the content analysis, all suggestions can be categorized into five main groups. The percentages in a frequency distribution of participants’ opinions are, in descending order, as follows: training courses on knowledge and skills (40.7%), law issues (20.3%), redundancy with physicians (13.2%), safety (6.0%), patient’s confidence (4.2%), and other recommendations (15.6%). The details of each topic are listed as follows.

### 3.1. Training Courses on Knowledge and Skills of Using BTX in Dentistry

A formal course recognized and accredited by the Thai Dental Council should be established and offered in an educational institution or faculty of dentistry. Adequate and accessible training courses should be provided. A license or certificate should be provided to dentists who pass some form of qualifying examination to ensure that they can use BTX in the correct and safe way to treat dental patients.

### 3.2. Law Issues

There should be a formal declaration of the law that covers the scope of treatment, procedures, access to training courses, organizations or places for training, and qualifications of dentists to use BTX. The Thai Dental Council should be able to issue an official license for BTX use in dentistry as well. In other words, there must be strict control of the use of BTX by dentists, including the standard procedure of BTX use, the distribution, and the advertising contents.

### 3.3. Redundancy with Physicians

The scope of the dentist’s work should not overlap with the physician’s one in terms of esthetics. For example, dentists should only use BTX to treat dental-related diseases or benefit dental treatment. For esthetic purposes, there must be limitations to the parts of facial structure and anatomy that dentists can treat.

### 3.4. Safety

There should be more research to support the idea that BTX can be used effectively in dental treatment. BTX should only be used when necessary. Any other alternatives should be applied before the decision to use BTX. The use of BTX must be performed with caution.

### 3.5. Patient Confidence

The fact that dentists can use BTX in treatment should be publicized. The advantages and disadvantages must be clarified so that the patient can decide for himself/herself.

## 4. Discussion

The primary objective of this study was to investigate the awareness and attitude of Thai dental practitioners towards the use of botulinum toxin (BTX) in dentistry. The survey sample was large enough to statistically represent the currently practicing dentists in Thailand. We found that about 80% of participants agreed to the use of BTX in their dental patients. However, the main barriers that kept the participants from using BTX in dentistry were their knowledge and experience in BTX application and related laws.

The result from Table 2 shows that most participants knew that BTX could be used to repair facial esthetics (gummy smile, masseteric hypertrophy) and treat bruxism. In contrast, more than half of dentists were unaware that BTX has other therapeutic benefits. This is consistent with the results that dentists may have knowledge of the mechanism of BTX acting on muscles but lack knowledge of BTX applications in other fields of dentistry [20]. A similar trend was also seen in the study by Al Hamdan et al. (2013) [21]. Most dentists in Riyadh were aware that BTX could be used for cosmetic purposes, of which wrinkle reduction was the most reported (73.7%) followed by treatment of a gummy smile (51.0%) [21]. Additionally, a study by Alfouzan and Mekkawy (2021) found that 88.6% of dental faculty members in Saudi Arabia thought that wrinkle reduction was the most common indication of BTX [22].

We investigated factors that might be associated with the opinion to use BTX in dentistry using the multiple logistic regression analysis and selected only those with a *p*-value less than 0.05 to show in Table 4. Interestingly, male participants had more chances to use the BTX in their dental patients than females, with the adjusted odds ratios of AOR = 2.16 and 95% confidence interval of 1.16–4.03. Although most of the participants were female (72.7%), the large difference in the number of male to female participants likely did not affect the main result of our study as both male and female dentists agreed to the use of BTX. The only difference was the percentage of participants in different genders who agreed with BTX use, i.e., 87.6% and 76.25% in male and female participants, respectively.

According to Table 4, the participants at ages 30–35 and under 30 years old were 2.49 and 2.22 times more likely to use BTX in dentistry than those over 35 years old, respectively. The interest in further specialization in restorative and esthetic dentistry also affected the opinion on the use of BTX with an AOR of 1.66 (95% CI of 1.01–2.71). Participants who had experience with BTX had a 2.75-fold increase in deciding to use BTX in dental patients compared to those who had never used BTX. One possibility was that participants with experience of using BTX had achieved satisfactory efficacy. This is in agreement with Polo (2008), who found that 88% of patients treated for gummy smile would like to repeat the BTX treatment in the future [23]. We also found that the participants with a high level of BTX knowledge were 2.88 times more likely to decide to use BTX than those at the low level. Al Hamdan et al. (2013) showed that, among 60% of their participants who refused to use BTX, 40% lacked the BTX knowledge and experience [21], which is consistent with our study. It is implied that gaining more knowledge about BTX may lead to an attitude that favors the BTX use in dentistry.

In the attitude section, we found an impressively high percentage of the participants (90%) agreed to the use of the BTX as a combination treatment for dental patients as it is thought to be beneficial to the patients. Almost all participants agreed that using BTX in dental patients would update knowledge (93.7%) and improve efficiency in treating dental patients (94.9%). It is clearly suggested that dentists have a positive attitude towards the use of BTX in dentistry. Furthermore, this is consistent with the high percentage (79.3%) of the participants who agreed to use BTX to treat dental patients when they are ready or have no obstacles. This indicates that the use of BTX in dentistry in Thailand would probably increase if the relevant difficulties are eliminated.

Despite very positive attitudes towards using BTX in dental patients, only 26 (out of 444, 5.86%) participants had ever provided BTX treatment for their patients. It is clearly indicated that BTX is not widely used by Thai dentists today. A similar situation happened in Riyadh, Saudi Arabia; only 1.2 percent of dentists used BTX to treat dental patients (2013) [21]. The latest study in 2021 still showed the same results; only 3% of dentists at the Faculty of Dentistry in Saudi Arabia had ever used BTX to treat dental patients [22]. Khalid et al. (2021) also found that only 3.51% of 199 dentists from Saudi Arabia and Pakistan used botulinum toxin for dental treatment [24].

The inconsistency between a positive attitude and the experience of using BTX in Thai dentists may be due to some difficulties in adopting BTX to treat dental patients. From question A3 in Table 3, the top ranking impediment was the law ambiguity regarding the scope of work of dentists, with an average score of 4.12. This ambiguity may be due to the fact that Thai dentists do not have enough knowledge of the law. According to Section 4 of the Dental Professional Act, B.E. 2537 (1994), dentists can perform an extraoral treatment if the disease or condition originates from dental disease, oral organ disease, jawbone, and facial bone disease associated with the jaw, including symptoms caused by the conditions mentioned above [25]. Therefore, Thai dentists are legally allowed to use BTX to treat disease only within the above scope [26]. It would be better to explicitly clarify and advertise the limited scope within which Thai dentists can work with BTX in the form of an official announcement to avoid any misunderstanding.

Lack of knowledge and experience is ranked second for preventing Thai dentists from using BTX, with a mean score of 4.04 (Table 3). As Thailand has not yet established a formally recognized course of BTX study, most Thai dentists do not have access to educational resources. In contrast, BTX training courses recognized by the dental council are offered in many countries. For example, the American Academy of Facial Aesthetics (AAFA) operates more than 50 courses per year by dividing the training courses into levels. These include the use of BTX for cosmetic purposes and the treatment of pain in the face and jaw [27]. The states of Massachusetts and New Jersey, USA, require dentists to pass the training course before treating patients with BTX [9]. Likewise, the New Zealand Dental Council issued a conclusion in 2017 that dentists must be trained to use BTX to treat patients, which is limited to orofacial complex and related organs [28]. Not only should the training courses be offered, but the Thai dental council should also establish rules or issue official training certificates to control the BTX use for both dentists and physicians. The clear scope of work and certified training courses will promote the use of BTX in dentistry and reduce redundancy in treatment with BTX of dentists and physicians.

Other difficulties with lower average scores in Table 3 include unknown sources of materials and equipment (3.22) and concerns of the patients (3.03). These obstacles might be diminished by the establishment of the training courses officially offered or certified by the Thai dental council.

### Limitations

Despite a clear result of positive attitudes of dental practitioners towards the use of BTX in dentistry, our study still has limitations and biases. The sample selection was achieved on a voluntary basis through a closed electronic discussion group (LINE messaging application operated by Line Corporation). It is possible that only socially engaging dentists were comfortable answering the questionnaires. Therefore, the responses might be biased towards similar populations of participants such as those at young ages. However, this might not be a crucial factor, as most of the participants at ages over 35 years old (67.3%) also agreed to the use of BTX in their dental patients, which is similar to the opinion of those younger than 35 years old.

## 5. Conclusions

We found that most Thai dentists realized the importance and had a high interest in the use of BTX in dentistry. Nevertheless, they were still concerned about obstacles in many areas, especially the law and quality control of the usage procedure. Only a few dentists were aware that Thailand has a law limiting the scope of using BTX in dentistry. The advertisement to promote the scope of legally using BTX is necessary to provide both Thai dentists and patients with enough knowledge of the related laws. Furthermore, official training courses and licenses certified by the Professional Council should be established to prevent problems or redundancy between dentists and physicians that might occur in the future. Further studies regarding the adverse effects of using BTX in dental patients and the cost-effectiveness of BTX treatment are desirable.

## Figures and Tables

**Table 1:** Demographics data of participants.

Characteristic	Total	Opinion on Using BTX in Dentistry		
	(*n* = 444)	Disagree (*n* = 92)	Agree (*n* = 352)	*p*-Value
	*n* (%)	*n* (%)	*n* (%)	
Gender				0.008 *
Male	121 (27.3%)	15 (12.4%)	106 (87.6%)	
Female	323 (72.7%)	77 (23.8%)	246 (76.2%)	
Age (mean age 32.7 years)				0.002 *
<30	167 (37.6%)	31 (18.6%)	136 (81.4%)	
30–35	173 (39.0%)	27 (15.6%)	146 (84.4%)	
>35	104 (23.4%)	34 (32.7%)	70 (67.3%)	
Specialty				0.133
No	160 (36.0%)	27 (16.9%)	133 (83.1%)	
Yes	284 (64.0%)	65 (22.9%)	219 (77.1%)	
Specialty field (n = 284) **				*0.339*
*Restorative and esthetic dentistry*	*169 (59.5%)*	*42 (24.9%)*	*127 (75.1%)*	
*Other specialists*	*115 (40.5%)*	*23 (20.0%)*	*92 (80.0%)*	
Interested in continuing education in the field of restorative and esthetic dentistry				0.032 *
Yes	218 (49.1%)	36 (16.5%)	182 (83.5%)	
No	226 (50.9%)	56 (24.8%)	170 (75.2%)	
Worksite				0.356
Rural	266 (59.9%)	57 (21.4%)	209 (78.6%)	
Urban	178 (40.1%)	35 (19.7%)	143 (80.3%)	
Affiliation				0.815
Private sector	125 (28.2%)	25 (20.0%)	100 (80.0%)	
Government	319 (71.8%)	67 (21.0%)	252 (79.0%)	
Years of practice				0.012 *
<20 years	402 (90.5%)	77 (19.2%)	325 (80.8%)	
≥20 years	42 (9.5%)	15 (35.7%)	27 (64.3%)	
Experience with botulinum toxin (BTX)				0.001 *
No	281 (63.3%)	72 (20.7%)	209 (79.3%)	
Yes	163 (36.7%)	20 (12.7%)	144 (87.7%)	
Experience with BTX (n = 163) ***				*0.352 ^F^*
*Have provided and received BTX treatment*	*23 (14.2%)*	*1 (4.3%)*	*22 (95.7%)*	
*Have provided BTX treatment*	*3 (1.8%)*	*0 (0.0%)*	*3 (100.0%)*	
*Have received BTX treatment*	*137 (84.0%)*	*19 (13.9%)*	*118 (86.1%)*	

* Statistically significant at *p* < 0.05. ^F^ Fisher’s exact test. ** The italics are results from only the participants with the dental specialties. *** The italics are results from only the participants with BTX experience.

**Table 2:** Background knowledge concerning the application of botulinum toxin (BTX) in dentistry.

Question	Total	Opinion on Using BTX in Dentistry		
	(*n* = 444)	Disagree (*n* = 92)	Agree (*n* = 352)	*p*-Value
	*n* (%)	*n* (%)	*n* (%)	
Which of the following problems can BTX help with?				
- Gummy smile				<0.001 *
Yes	311 (70.0%)	46 (14.8%)	265 (85.2%)	
No	133 (30.0%)	46 (34.6%)	87 (65.4%)	
- Masseteric hypertrophy				0.001 *
Yes	297 (66.9%)	48 (16.2%)	249 (83.8%)	
No	147 (33.1%)	44 (29.9%)	103 (70.1%)	
- Bruxism				0.006 *
Yes	334 (75.2%)	59 (17.7%)	275 (82.3%)	
No	110 (24.8%)	33 (30.0%)	77 (70.0%)	
- Mandibular spasm				0.182
Yes	235 (52.9%)	43 (18.3%)	192 (81.7%)	
No	209 (47.1%)	49 (23.4%)	160 (76.6%)	
- Myofascial pain				0.513
Yes	221 (49.8%)	43 (19.5%)	178 (80.5%)	
No	223 (50.2%)	49 (22.0%)	174 (78.0%)	
- Promoting prosthodontic treatment				<0.001 *
Yes	151 (34.0%)	16 (10.6%)	135 (89.4%)	
No	293 (66.0%)	76 (25.9%)	217 (74.1%)	
- Promoting orthodontic treatment				0.010 *
Yes	136 (30.6%)	18 (13.2%)	118 (86.8%)	
No	308 (69.4%)	74 (24.0%)	234 (76.0%)	
- Facilitating differential diagnosis				0.012 *
Yes	118 (26.6%)	15 (12.7%)	103 (87.3%)	
No	326 (73.4%)	77 (23.6%)	249 (76.4%)	
- Trigeminal neuralgia				0.947
Yes	105 (23.6%)	22 (21.0%)	83 (79.0%)	
No	339 (76.4%)	70 (20.6%)	269 (79.4%)	
- Sialorrhea				0.110
Yes	67 (15.1%)	9 (13.4%)	58 (86.6%)	
No	377 (84.9%)	83 (22.0%)	294 (78.0%)	
Levels of knowledge about the use of BTX in dentistry				<0.001 *
- Low (1–5 correct answers)	322 (72.5%)	80 (24.8%)	242 (75.2%)	
- High (6–10 correct answers)	122 (27.5%)	12 (9.8%)	110 (90.2%)	

* Statistical significance at *p* < 0.05.

**Table 3:** The attitude of 444 Thai dental practitioners towards using BTX in dentistry.

**Topics**	**Strongly Disagree** ***n* (%)** **(1)**	**Somewhat Disagree** ***n* (%)** **(2)**	**Not Sure** ***n* (%)** **(3)**	**Somewhat Agree** ***n* (%)** **(4)**	**Strongly Agree** ***n* (%)** **(5)**	**Favorable Score** ***n* (%)** **(4,5)**	**Mean ± SD**
A1. Do you agree that the botulinum toxin should be used as a combination treatment for patients who receive dental services in various fields such as occlusion dentistry, pain control, and esthetic dentistry?	3 (0.7%)	3 (0.7%)	40 (9.0%)	163 (36.7%)	235 (52.9%)	398 (89.6%)	4.2 ± 0.7
**Topics**	**Very Unbeneficial** ***n* (%)** **(1)**	**Somewhat Unbeneficial** ***n* (%)** **(2)**	**Not Sure** ***n* (%)** **(3)**	**Somewhat Beneficial** ***n* (%)** **(4)**	**Very Beneficial** ***n* (%)** **(5)**	**Favorable Score** ***n* (%)** **(4,5)**	**Mean ± SD**
A2. Would it be beneficial to the patients if a dentist used botulinum toxin for patients receiving dental services?	1 (0.2%)	2 (0.5%)	51 (11.5%)	131 (29.5%)	259 (58.3%)	390 (87.8%)	4.2 ± 0.6
**Topics**	**Not a Considerable Obstacle** ***n* (%)** **(1)**	**Not an Obstacle** ***n* (%)** **(2)**	**Not Sure** ***n* (%)** **(3)**	**An Obstacle** ***n* (%)** **(4)**	**A Considerable Obstacle** ***n* (%)** **(5)**	**Favorable Score** ***n* (%)** **(4,5)**	**Mean ± SD**
A3. Are the following factors obstacles to the use of botulinum toxin in dental patients?							
A3.1 The ambiguity of law regarding the scope of work of dentists	4 (0.9%)	13 (2.9%)	73 (16.4%)	164 (36.9%)	190 (42.8%)	354 (79.7%)	4.1 ± 0.8
A3.2 Lack of knowledge and experience	11 (2.5%)	31 (7.0%)	38 (8.6%)	152 (34.2%)	212 (47.7%)	364 (81.9%)	4.0 ± 1.0
A3.3 Lack of additional educational resources for dentists	8 (1.8%)	41 (9.2%)	43 (9.7%)	145 (32.7%)	207 (46.6%)	352 (79.3%)	4.0 ± 1.0
A3.4 Redundancy in the treatment between physicians and dentists	23 (5.2%)	77 (17.3%)	82 (18.5%)	99 (22.3%)	163 (36.7%)	262 (59.0%)	3.5 ± 1.1
A3.5 Unknown sources of materials and equipment	31 (7.0%)	51 (11.5%)	86 (19.4%)	109 (24.5%)	167 (37.6%)	276 (62.1%)	3.2 ± 1.1
A3.6 Distrust or deemed unacceptable by the patient of the dentist	23 (5.2%)	27 (6.1%)	127 (28.6%)	133 (30.0%)	134 (30.2%)	267 (60.2%)	3.0 ± 1.0
A3.7 Increasing patient costs	32 (7.2%)	33 (7.4%)	123 (27.7%)	126 (28.4%)	130 (29.3%)	256 (57.7%)	3.0 ± 1.1
A3.8 Increasing duration of treatment	23 (5.2%)	59 (13.3%)	75 (16.9%)	124 (27.9%)	163 (36.7%)	287 (64.6%)	2.6 ± 1.1
A3.9 Inappropriate treatment location	28 (6.3%)	85 (19.1%)	86 (19.4%)	99 (22.3%)	146 (32.9%)	245 (55.2%)	2.6 ± 1.2
**Topics**	**Strongly Disagree** ***n* (%)** **(1)**	**Somewhat Disagree** ***n* (%)** **(2)**	**Not sure** ***n* (%)** **(3)**	**Somewhat agree** ***n* (%)** **(4)**	**Strongly Agree** ***n* (%)** **(5)**	**Favorable Score** ***n* (%)** **(4,5)**	**Mean ± SD**
A4. How strongly do you agree with the use of botulinum toxin in dental patients?							
A4.1 Updating the knowledge	2 (0.5%)	6 (1.4%)	20 (4.5%)	185 (41.7%)	231 (52.0%)	416 (93.7%)	4.3 ± 0.7
A4.2 Improving efficiency in treating dental patients	2 (0.5%)	2 (0.5%)	19 (4.3%)	197 (44.4%)	224 (50.5%)	421 (94.9%)	4.4 ± 0.6
A4.3 Increasing income	22 (5.0%)	51 (11.5%)	81 (18.2%)	135 (30.4%)	155 (34.9%)	290 (65.3%)	3.5 ± 1.1
A5. Will you agree to use botulinum toxin to treat your dental patients when you are ready or have no obstacles?	3 (0.7%)	5 (1.1%)	84 (18.9%)	135 (30.4%)	217 (48.9%)	352 (79.3%)	4.1 ± 0.8

**Table 4:** Multivariate logistic regression analysis of factors associated with opinion on the use of botulinum toxin in dental patients (*n* = 444).

Factors	Opinion on Use of Botulinum Toxin in Dentistry				
	Unadjusted OR	95% CI	Adjusted OR	95% CI	*p*-Value
Gender					
Female	1.00	Reference	1.00	Reference	
Male	2.21	1.22–4.02	2.16	1.16–4.03	0.016 *
Age (years)					
>35	1.00	Reference	1.00	Reference	
30–35	2.63	1.47–4.69	2.49	1.35–4.57	0.003 *
<30	2.13	1.21–3.75	2.22	1.22–4.02	0.009 *
Interest in continuing education in restorative and esthetic dentistry					
No	1.00	Reference	1.00	Reference	
Yes	1.66	1.04–2.66	1.66	1.01–2.71	0.044 *
Experience with botulinum toxin					
No	1.00	Reference	1.00	Reference	
Yes	2.46	1.44–4.22	2.75	1.56–4.85	<0.001 *
Level of knowledge about botulinum toxin in dentistry					
Low	1.00	Reference	1.00	Reference	
High	3.03	1.59–5.79	2.88	1.48–5.61	0.002 *

* Statistical significance at *p* < 0.05.

## Data Availability

The data presented in this study are available upon request from the corresponding author.
