# Correction: Laorpipat et al. Attitude of Thai Dental Practitioners towards the Use of Botulinum Toxin in Dentistry. *Int. J. Environ. Res. Public Health* 2022, *19*, 1878

**DOI:** 10.3390/ijerph20247165

**Published:** 2023-12-12

**Authors:** Sasi Laorpipat, Pornpoj Fuangtharnthip, Suraphong Yuma, Chanita Tantipoj

**Affiliations:** 1 Department of Advanced General Dentistry, Faculty of Dentistry, Mahidol University, Ratchathewi, Bangkok 10400, Thailand; sasi.lao@mahidol.ac.th (S.L.); pornpoj.fun@mahidol.ac.th (P.F.); 2 Department of Physics, Faculty of Science, Mahidol University, Ratchathewi, Bangkok 10400, Thailand; suraphong.yum@mahidol.edu

## Text Correction

There was an error in the original publication [1]. It was documented that “This study is a cross-sectional study carried out from July 2020 to April 2021 and was approved by the Ethics Committee of Human Research, Faculty of Dentistry and Faculty of Pharmacy, Mahidol University, Bangkok, Thailand (no. MU-DT/PY-IRB 2020/038.2608).”

A correction has been made to *2. Materials and Methods*, *Paragraph 1*:

“This study is a cross-sectional study approved by the Ethics Committee of Human Research, Faculty of Dentistry and Faculty of Pharmacy, Mahidol University, Bangkok, Thailand (no. MU-DT/PY-IRB 2020/038.2608). Data collection was carried out from December 2020 to April 2021.”

The authors state that the scientific conclusions are unaffected. This correction was approved by the Academic Editor. The original publication has also been updated.
